# Case report: A case of isolated cardiac sarcoidosis diagnosed by multimodal imaging and endomyocardial biopsy

**DOI:** 10.3389/fcvm.2022.993024

**Published:** 2022-10-14

**Authors:** Yongling Wa, Xiaowei Niu, Jizhe Xu, Gaxue Jiang, Sixiong Hu, Ming Bai

**Affiliations:** Department of Cardiology, First Hospital of Lanzhou University, Lanzhou, China

**Keywords:** cardiovascular imaging (CARD), isolated cardiac sarcoidosis, myocardial endocardial biopsy, multimodal imaging, CMR (cardiovascular magnetic resonance), FDG (18F-fluorodeoxyglucose)-PET/CT

## Abstract

Due to its low incidence, isolated cardiac sarcoidosis (ICS) is often missed or misdiagnosed. Herein, we describe a case of ICS in a 52-year-old male patient. Advanced imaging, including cardiac magnetic resonance (CMR) and fluorine-18 fluorodeoxyglucose positron emission tomography (FDG-PET), could not only screen high-risk patients for establishing diagnosis, but also guide endomyocardial biopsy (EMB) for improving cardiac disease detection rate. This case highlights the importance of multimodal imaging for screening and necessity of EMB for diagnosis.

## Introduction

Sarcoidosis is a systemic disease that can occur in various organs. Although the most common clinical manifestation in > 90% of patients with sarcoidosis is enlargement of the hilar and mediastinal lymph nodes, circular erythema of the skin is the main symptom in some patients. However, some patients (approximately 5%) also have heart involvement called cardiac sarcoidosis (CS) that can develop in any part of the heart. The three most common clinical manifestations of CS are atrioventricular block, ventricular arrhythmia, and heart failure. Some studies reported that a small number of primary lesions in patients with sarcoidosis originate from the heart: isolated cardiac sarcoidosis (ICS). The definition of ICS was first established in the CS guidelines proposed by the Japanese Circulation Society (1–3). This case is unique in that it provides a preliminary diagnosis of ICS through multimodal imaging combined with identifying clinical manifestations. An accurate endomyocardial biopsy (EMB) was performed under the guidance of imaging to successfully make diagnosis (Table 1).

**TABLE 1:** The patient’s time line.

Time line	
2021.7 First hospitalization	The patient was admitted due to chest tightness and palpitation, electrocardiogram showed complete right bundle branch block, No obvious abnormalities were found on coronary angiography. CMR imaging revealed abnormal signals in the right ventricular surface of the ventricular septum, and significant LGE. Patient underwent cardiac catheter ablation and drug therapy
2022.4 Second hospitalization	Repeat CMR imaging showed LGE were aggravated. FDG-PET showed significant metabolic increase in ventricular septum and right ventricle, and no abnormal metabolic lesions were observed in other organs of the body.
2022.6 Third hospitalization	Patient received the last CMR to accurately EMB. It was eventually diagnosed as ICS, received an ICD and drug therapy.
2022.8 Follow-up	During 2 months after discharge, the patient had no symptoms of palpitations. FDG-PET showed that the area of increased glucose metabolism in the original lesion was reduced, indicating the effectiveness of immunosuppressive therapy.

CMR, cardiac magnetic resonance; FDG-PET, Fluorine-18 fluorodeoxyglucose positron emission tomography; EMB, endomyocardial biopsy.

## Case description

A 52-year-old man presented to our hospital with palpitations and dizziness. Physical examination on admission did not show any skin or mucosal damage or superficial lymph node enlargement. Laboratory test results showed only a slight increase in troponin levels (0.032 ng/ml). In addition, electrocardiography (ECG) showed ventricular premature contractions and complete right bundle branch block [left ventricular ejection fraction (LVEF) % = 65%].

**FIGURE 1:**
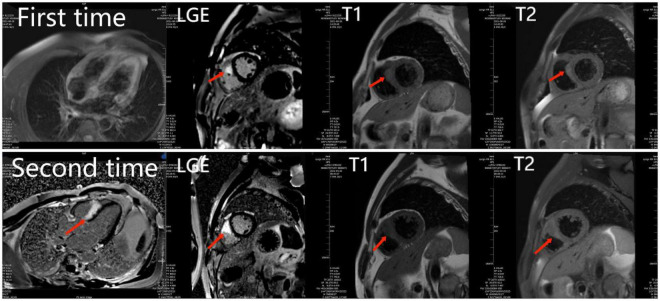
The first and second cardiac magnetic resonance show abnormal signals and LGE in the right ventricular surface of the ventricular septum. And the second time LGE was more severe than the first time.

Eleven months prior, the patient experienced unexplained intermittent palpitations, with dizziness, fatigue, chest tightness, and shortness of breath. He was admitted to a local hospital. Twenty-four hour Holter monitoring in the patient showed frequent ventricular extrasystoles, paroxysmal ventricular tachycardia, and right bundle branch block. Echocardiography revealed LVEF of 60% with no obvious abnormalities in cardiac structure or function. Cardiac magnetic resonance (CMR) imaging revealed abnormal signals in the right ventricular surface of the ventricular septum, and late gadolinium enhancement (LGE) (Figure 1). Based on these findings, the patient was diagnosed with multifocal ventricular tachycardia. Therefore, the patient underwent cardiac catheter ablation and received bisoprolol (5 mg/d), amiodarone (0.2 g/d).

**FIGURE 2:**
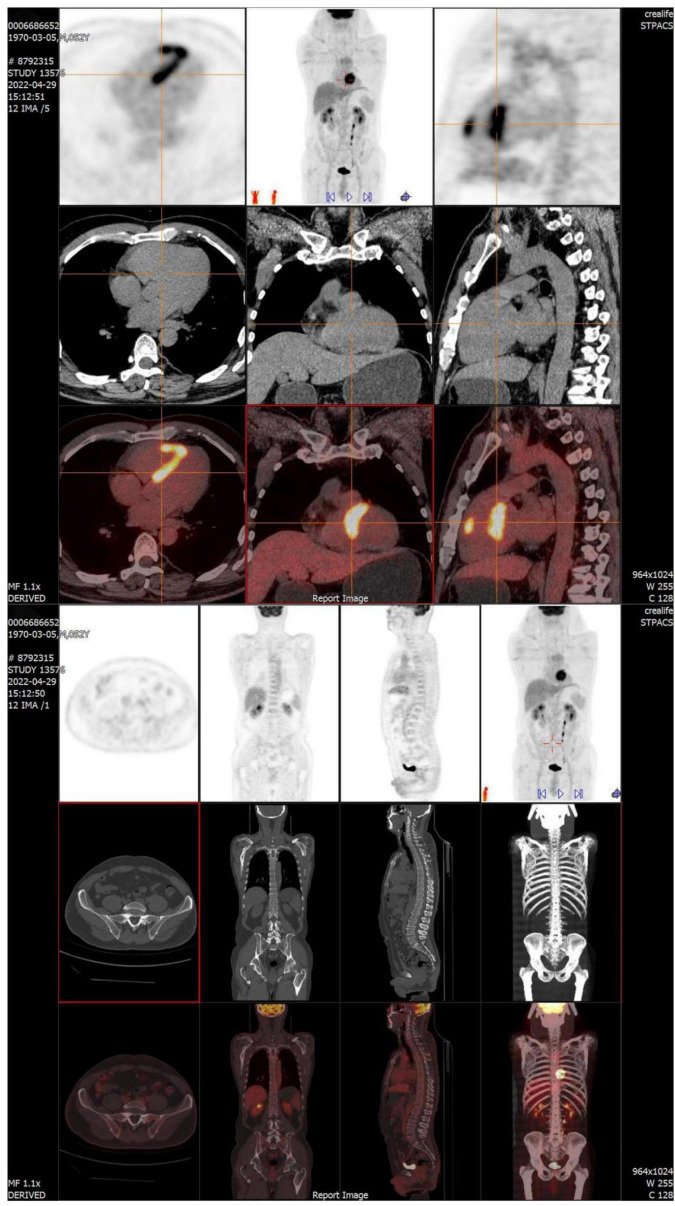
FDG-PET reveals intense multifocal cardiac uptake in the interventricular septum and right ventricle, and no abnormal lesions are found in other parts of the body. FDG-PET, Fluorine-18 fluorodeoxyglucose positron emission tomography.

Surgery and medication did not improve the patient’s symptoms within the following 7 months. In local hospital repeat CMR imaging showed that LGE was aggravated (Figure 1). Fluorine-18 fluorodeoxyglucose positron emission tomography (FDG-PET) demonstrated intense multifocal cardiac uptake in the interventricular septum and right ventricle without abnormal lesions in other parts of the body (Figure 2). Adenosine technetium-99m (99mTc)-sestamibi myocardial perfusion imaging showed that blood perfusion was decreased in the middle and basal segments of the ventricular septum. The doctor considered that arrhythmia might be caused by cardiac tumor, and suggested that the patient should undergo cardiac biopsy in our hospital.

After admission, based on the patient’s imaging findings and symptoms, we considered a possible diagnosis of: CS, myocarditis, hypertrophic cardiomyopathy, or myocardial amyloidosis. CS can invade any part of the myocardium. However, transmural involvement of the left and right ventricular walls is the most common. The inflammatory phase of CS is characterized by myocardial granulomatous infiltration, inflammation, and edema. This can lead to local thickening and abnormal movement of the heart muscle. Although both CS and myocarditis manifest cardiac inflammation, myocarditis mostly involves the lateral wall of the heart, whereas CS mostly involves the septum (4). In patients with CS, myocardial granulomatous infiltration can lead to thickening of the myocardial wall, morphologically similar to hypertrophic cardiomyopathy. However, in this case, abnormal FDG-PET uptake foci were observed. Moreover, ECG showed ventricular arrhythmia, and hypertrophic cardiomyopathy was not detected. The most typical imaging features of cardiac amyloidosis are extensive LGE, most prominent in the subendocardial layer. In addition, in CS, subepicardial LGE is observed.

**FIGURE 3:**
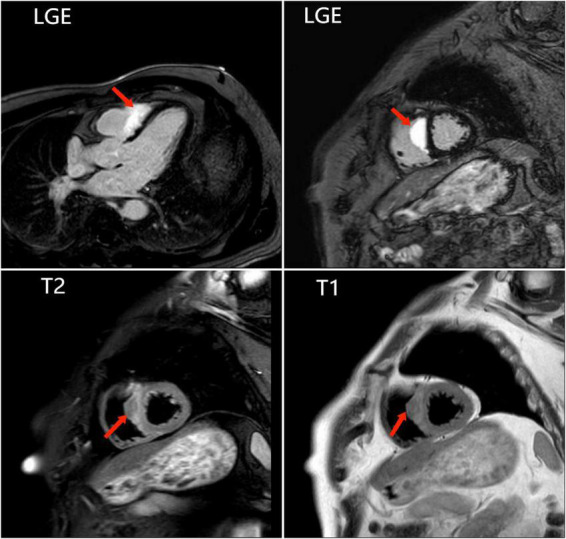
The last cardiac magnetic resonance is used for accurately locating the lesion and improving the success rate of the endomyocardial biopsy. Images show abnormal signals in the right ventricular surface of the ventricular septum, and significant LGE.

**FIGURE 4:**
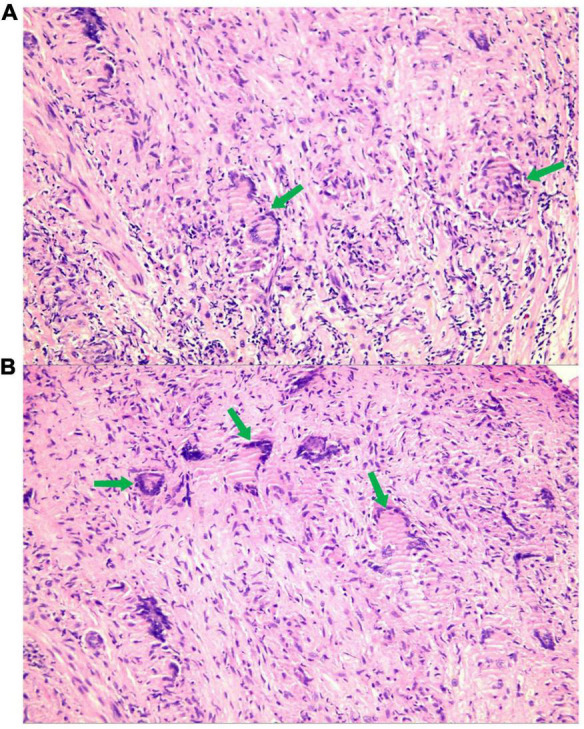
A large number of epithelioid nodules composed of epithelioid cells, multinucleated giant cells [**A,B** (green arrow)], and lymphocytes are found in the myocardium and endocardium.

To further clarify the cause of arrhythmia, we performed an EMB. To accurately locate the lesion and improve the success rate of the EMB, the patient underwent the last CMR (Figure 3). Using CMR imaging guidance, EMB was taken from interventricular septum tissue of the right ventricle. EMB revealed a large number of epithelioid nodules composed of epithelioid cells, multinucleated giant cells, and lymphocytes in the myocardium and endocardium. However, no necrotic tissue was found on acid-fast staining (–) (Figure 4). Based on the clinical manifestations, imaging data, and pathological features, we diagnosed the patient with ICS. Thereafter, the patient received dual-chamber implantable cardioverter defibrillator to prevent sudden death, while prednisone (35 mg/d) was administered to treat sarcoidosis. Additionally, spironolactone (20 mg/d) and daglitazine (10 mg/d) were administered to delay ventricular remodeling.

**FIGURE 5:**
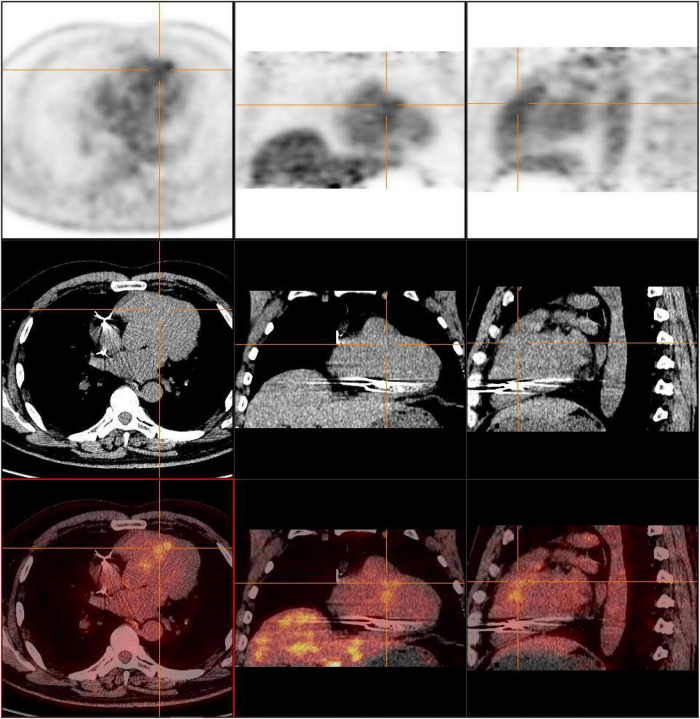
FDG-PET showed that the area of increased glucose metabolism in the original lesion was reduced, indicating the effectiveness of immunosuppressive therapy.

During 2 months after discharge, the patient had no symptoms of palpitations. The 24-h Holter monitoring showed frequent premature ventricular contractions without further ventricular tachycardia. FDG-PET showed that the area of increased glucose metabolism in the original lesion was reduced, indicating the effectiveness of immunosuppressive therapy (Figure 5).

## Discussion

Due to the low incidence and inconsistent diagnostic methods of ICS, it is often missed or misdiagnosed. However, multimodal imaging evaluation is of great significance to guide diagnosis of CS. A Japanese study indicated that approximately 26.8% of patients with CS could be diagnosed with ICS (5). Some studies have shown that patients with ICS might have poor prognosis and are more likely to develop heart failure and other adverse cardiac events (6). Therefore, early diagnosis of CS using multimodal imaging is useful for achieving a favorable patient prognosis. Advanced cardiac imaging of CS includes CMR and FDG-PET. A meta-analysis by Zhang et al. showed that CMR imaging had 95% sensitivity and 92% specificity for detection of CS (7). On the one hand, FDG-PET can be used for detecting myocardial inflammation and metabolism of lesions caused by granulomatous infiltration in patients with CS. However, FDG-PET can detect systemic lesions outside the heart and help identify other organs affected by sarcoidosis. CMR focuses on examining deconstruction and perfusion of the heart and can directly reflect exercise ability and degree of fibrosis of the heart. FDG-PET focuses more on the degree of inflammation and metabolism of lesions. The complementary advantages of the two methods can help better diagnose CS, which is the significance of multimodal imaging examinations.

Although some researchers advocate CS diagnosis through clinical manifestations and imaging examination, EMB is the gold standard for CS diagnosis. Owing to the uneven distribution of non-caseous granulomas in the myocardial endocardium, the positive predictive accuracy of EMB is low. In our case, to accurately obtain tissue from the lesion, we used CMR to locate the lesions and thus, obtained positive results. Some studies have reported that the positive predictive value of EMB could reach 89% (8) when the electrolytic voltage profile is consistent with that of the CMR image. Therefore, regarding CS, advanced imaging cannot only screen high-risk patients to assist diagnosis but also guide EMB to improve the detection rate. Based on our experience, advanced imaging examination should be improved in time to prevent missed diagnosis in patients with the following conditions: First, unexplained palpitations, syncope, decreased ejection fraction, or previous heart failure. Second, unexplained left or right bundle branch block; second or third degree atrioventricular block; atrial arrhythmia; and ventricular arrhythmia occur. Third, unexplained wall thickness and wall motion abnormalities are observed on echocardiography.

Corticosteroid immunosuppressants are currently the first-line treatment of sarcoidosis as they improve cardiac remodeling and effectively relieve symptoms when used as early as possible after CS diagnosis. Of note, CMR and FDG-PET scans can also be used for evaluating the efficacy of immunosuppressive therapy in patients with CS.

Based on the findings of our case, due to lack of specific biomarkers for detecting CS and ICS, diagnosis is still very difficult. Multimodal imaging examination should be used more frequently in patients with suspected CS or having high-risk factors for CS. Timely and accurate diagnosis has important clinical significance for better follow-up, treatment, and prognosis of patients.

## Data availability statement

The original contributions presented in this study are included in the article/supplementary material, further inquiries can be directed to the corresponding author.

## Ethics statement

The studies involving human participants were reviewed and approved by the Clinical Research Ethics Committee, First Hospital of Lanzhou University. The patients/participants provided their written informed consent to participate in this study. Written informed consent was obtained by the patients/participants for the publication of this case study.

## Author contributions

YW and XN contributed to manuscript writing. JX, GJ, SH, and MB contributed to concept design, manuscript editing, and revision. All authors contributed to the article and approved the submitted version.
